# Using Shifts in Amino Acid Frequency and Substitution Rate to Identify Latent Structural Characters in Base-Excision Repair Enzymes

**DOI:** 10.1371/journal.pone.0025246

**Published:** 2011-10-06

**Authors:** Ramiro Barrantes-Reynolds, Susan S. Wallace, Jeffrey P. Bond

**Affiliations:** Department of Microbiology and Molecular Genetics, University of Vermont, Burlington, Vermont, United States of America; National Institute on Aging, United States of America

## Abstract

Protein evolution includes the birth and death of structural motifs. For example, a zinc finger or a salt bridge may be present in some, but not all, members of a protein family. We propose that such transitions are manifest in sequence phylogenies as concerted shifts in substitution rates of amino acids that are neighbors in a representative structure. First, we identified rate shifts in a quartet from the Fpg/Nei family of base excision repair enzymes using a method developed by Xun Gu and coworkers. We found the shifts to be spatially correlated, more precisely, associated with a flexible loop involved in bacterial Fpg substrate specificity. Consistent with our result, sequences and structures provide convincing evidence that this loop plays a very different role in other family members. Second, then, we developed a method for identifying latent protein structural characters (LSC) given a set of homologous sequences based on Gu's method and proximity in a high-resolution structure. Third, we identified LSC and assigned states of LSC to clades within the Fpg/Nei family of base excision repair enzymes. We describe seven LSC; an accompanying Proteopedia page (http://proteopedia.org/wiki/index.php/Fpg_Nei_Protein_Family) describes these in greater detail and facilitates 3D viewing. The LSC we found provided a surprisingly complete picture of the interaction of the protein with the DNA capturing familiar examples, such as a Zn finger, as well as more subtle interactions. Their preponderance is consistent with an important role as phylogenetic characters. Phylogenetic inference based on LSC provided convincing evidence of independent losses of Zn fingers. Structural motifs may serve as important phylogenetic characters and modeling transitions involving structural motifs may provide a much deeper understanding of protein evolution.

## Introduction

For many protein families the sequences of hundreds of members, as well as the high-resolution structure of at least one member, are available. It is clear from inspection of those data sets that structural features vary during protein family evolution even when the overall fold is conserved. Such variation is reflected in amino acid substitutions that are correlated as well as substitution rates that are inhomogeneous, that is, sites exhibit heterotachy [1]. However, the most commonly applied models of protein sequence evolution do not capture these features. Our goal is to use explicit identification of transitions between states of structural characters for studying the evolution of protein sequence, structure, and function.

Amino acid substitutions reflect constraints imposed by the structural context of amino acids along reaction paths. In the case of base-excision repair proteins, first-shell residues interact directly with DNA backbone, to functional groups specific to a damage, or the opposite base. Substitutions of such amino acids are rare. Multiple second or third-shell amino acids may interact directly with first or second-shell residues respectively [2]. Amino acids in the second or third shell typically vary more quickly than first shell amino acids and might vary in concert, perhaps reflecting the existence of multiple ways to position a first shell residue. Concerted transitions involving such residues are nonetheless more rare than most other sequence substitutions. As such they may serve as important phylogenetic characters that vary more slowly than nucleotide or amino acid characters but more rapidly than catalytic residues or domain structure and thus aid phylogenetic inference related to distant times.

Many computational approaches [3], [4], [5], [6], [7], [8], [9], [10], [11], [12], [13], [14], [15], [16], [17], [18], [19], [20] try to identify amino acid sequence positions that are under functional evolutionary constraints (functional sites). These methods differ in that they may use structural information, employ models based on information theory, phylogenetic trees, energetic calculations, or incorporate information about amino acid chemical properties.

In addition, other methods identify shifts between conservation patterns that result in subfamily-specific sites. These are functional sites within a group of homologous protein subfamilies that confer the corresponding difference in function or structure. Most of these methods take advantage of the fact that the subfamily-specific sites will vary in their rate of evolution and/or tolerated amino acid frequencies between the paralogs. Heterotachous sites need not be related to subfamily-specific functions [1]. Gu and coworkers [21], [22], [23], [24], [25] developed a method [26] for finding subfamily-specific sites based on the idea that functional sites can be identified by changes in the evolutionary rate or the biochemical nature of the amino acids (type I and type II changes respectively). Other methods [3], [15], [27], [28], [29], [30], [31], [32], [33], [34], [35] have also been developed to find the subfamily specific sites and in some cases the subfamilies themselves.

It is understood that residues in a protein do not work in isolation but rather within a context as part of a cooperating system [36] that positions the substrate and residues required for binding and catalysis. Salt-bridges, zinc-fingers and catalytic units work together in order for the protein to function. Structural information can be used to identify conserved regions of the protein [37], and several authors have taken advantage of that. Panchenko et al. [38] identified functional sites by taking into account the conservation between structurally neighboring amino acids; Landgraf et al. [39] devised a method to identify functional clusters: groups of amino acids with some degree of conservation relative to the rest of the protein.

Studies have found evidence that stabilizing residues are more likely to be conserved and cluster structurally [40]. Approaches to establish statistical significance of clustering of functional divergence sites have been done on evolutionary trace sites [41], finding that they do form structural clusters. The sites found by the evolutionary trace method are sites that are highly conserved within each subfamily, but different between them. Other approaches have also recognized the structural cooperativity of important amino acids, and focused on conserved structural clusters among protein binding sites [42], on protein-protein interaction interfaces and their evolution [43], [44], and on conserved clusters of a single subfamily [45], [46], [47]. No approach has looked at the formation of structural clusters of rate-shift sites, nor has any study investigated using those clusters as characters phylogenetic inference.

The cooperating nature of amino acids is latent, or not immediately apparent, in the multiple sequence alignment. Therefore, we would like to extend the notion of functional and subfamily specific sites to latent structural characters (LSCs). We reasoned that subfamily-specific sequence/structure motifs could be identified by combining sequence-based identification of changes in selection pressure with information about proximity of amino acids in space. An LSC is then informally defined as a set of amino acids that are near each other in the protein structure and exhibit concerted changes in selection pressure. Our work is distinct from that of Gu and coworkers in that we calculate changes in amino acid frequency and substitution rate to explicitly infer shifts in the selection pressure on sets of amino acids based on a high-resolution structure. Our emphasis on detection of groups of neighboring amino acids that change concertedly in selection pressure lies at the heart of the novelty of our approach and reflects the assumption that amino acids do not work in isolation but rather cooperate in function. Our results can be easily viewed in three dimensions on the accompanying website (http://proteopedia.org/wiki/index.php/Fpg_Nei_Protein_Family), which intends to serve as a repository of information on LSCs in the Fpg/Nei protein family.

Genome integrity affects survival of cells and the organism and several pathways have evolved for protection against damages [48], [49], [50], [51]. The main protection against endogenous oxidative DNA damage (such as reactive oxygen species produced by metabolism) is the base excision repair system [52], [53], [54], [55], [56]. Its association with human disease and aging (for review see [57] and 47–49) is consistent with its importance. The Fpg/Nei base-excision repair family recognizes a wide range of DNA damages [58], [59], [60], [61], [62], [63], [64], [65], [66]. Its phylogeny is not well understood, presumably because their ancient origin makes phylogenetic inference difficult [58], [65], furthermore, its core domain is not related to any other known protein family. Organisms vary not only in the kinds of damages recognized by their Fpg/Nei enzymes, but also by their number of homologs: the actinomycetes phyla has four different paralogs, the eukaryotes have three, and proteobacteria two. With respect to substrate discrimination, we have some understanding on bacterial Fpg [67], [68], [69] and Escherichia coli Nei [70] but none on the other orthologous clades (see proteopedia page for substrate specificity information). Therefore, given the functional importance, unknown phylogeny, and the fact that the structural conservation amongst its members is high [71] we decided to apply our methods to this family.

We first showed how a group of rate-shift sites cluster structurally in the enzyme and how these sites cooperate to perform an important functional role. We then identified seven LSCs in this protein family, including both familiar structural features and those that have not previously been discussed. We propose how the amino acid roles between states of these characters relate to each other (for example, compensation) and how they are distributed in the phylogeny. We have also used them to resolve previously unresolved deep branches of the family. We found that the majority of amino acids exhibit a statistically significant change in amino acid propensities or substitution rates, presumably reflecting surprising fluidity of the interactions that stabilize protein structures. Finally, we found substantial variation in overall evolutionary substitution rates among homologous subfamilies, presumably a result of changing functional roles. We found that studying LSC, rather than individual amino acids, as well as focusing on events such as changes in rate or amino acid frequency rather than individual substitutions, can shed new light on evolution and function.

## Results

### Type I transitions are associated with a change in the mechanism of substrate recognition

**Figure 1:**
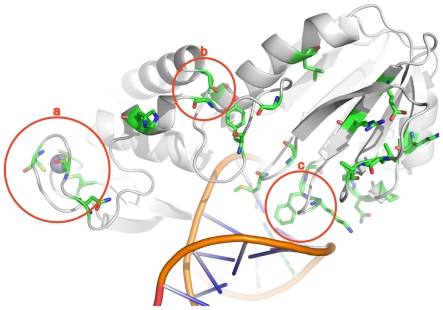
Example of structural clustering of type I sites. Type I sites, amino acids that shift in substitution rate among two clades (BaFpg1, and PFNei), are colored in green in the B. stearothermophilus MutM structure [67] (1R2Y). Three structural clusters (LSCs) are shown, the (a) zinc finger (BaFpg1), zincless finger (PFNei); b) two highly conserved glycines on Fpg which mark the beginning and end of the recognition loop, and which have a higher rate on PFNei, suggesting that the loop does not perform the same role in recognition and c) a triad that stabilizes the DNA and the opposite base which allows for more variability on PFNei.

For one quartet we rejected (p = 0.002) the null hypothesis that the type I transitions are not spatially correlated. More precisely, we permuted edge assignments of type I transitions occurring along the BaFpg1-PFNei or AcNei1-AcNei2 edges of the quartet and employed a test statistic reflecting amino acid proximity (see the Methods section). Inspection of the type I transitions suggests that the spatial association results from the fact that many of them belong to the βF-α10 loop (Figure 1). The βF-α10 loop plays a critical role in substrate recognition of 8-oxoG in BaFpg1 by making important hydrogen bonds with O6, N1, N7 and N2 of 8-oxoG [67]. BaFpg1 discriminates between 8-oxoG and G by exploiting the difference in the protonation state of N7 caused by the extra carbonyl group in 8-oxoG [67]. FapyG recognition is accomplished by the same loop, but in a strikingly different way [68].

The role of this loop is unknown in every clade except BaFpg1. It corresponds to a helix in the human Neil1 crystal structure [65], a much shorter loop in Mimivirus [72], and a disordered region in the bound EcoNei structure [70]. In particular, aligned regions in other clades contain gaps. This observation would appear to validate our result, that is, that the βF-α10 loop plays an important role in substrate specificity in BaFpg1 that it does not play in other clades.

Our observations suggest treating the βF-α10 loop as one structural character. Just as type I amino acids are identified after quantifying substitution counts at one aligned position relative to the number of substitutions in the remainder of the protein, a change in the summary substitution rate of a region relative to the rest of the protein may indicate a change in its role. We rejected the null hypothesis (Table 1) that the mean substitution rate in the BaFpg1 recognition loop is the same as that of the remainder of the enzyme (p<0.05; it evolves more slowly) and that the distribution of mutation rates in this loop is equal to the distribution of rates in the remainder of the protein (p<0.004). Notably the aligned region appears to evolve more rapidly than the remainder of the protein in other clades. Based on a likelihood ratio test the substitutions in the βF-α10 recognition loop vary among the nine clades (p<10^−15^; the BaFpg1 substitution rate is smaller than all but the PFNei). Again, these results suggest a concerted change that involves many amino acids in the βF-α10 loop that is reflected in their substitution rates.

**Table 1:** Comparison of tree lengths and hypothesis testing of randomness of the loop on all subclades.

Subfamily	Tree Length	Rate (loop)	Rate (remainder)	p-value (location)	p-value (distribution)
MeNeil1	13.6	0.88	1.00	0.32	0.64
MeNeil2	7.4	1.43	0.98	0.95	0.23
MeNeil3	6.1	0.83	1.00	0.22	0.33
BaFpg1	41.9	0.78	1.04	0.044	0.0039
BaFpg2	18.1	1.05	1.10	0.42	0.054
PFNei	16.8	0.69	1.02	0.054	0.060
AcNei1	19.2	0.98	1.08	0.30	0.027
AcNei2	18.2	1.46	1.07	0.97	0.77
PrNei	7.6	0.96	1.03	0.38	0.49

However, we note that the βF-α10 loop contains gaps in other clades. It is our expectation that that our combination of PAML and Gu's method provides for identification of substantial changes in selection pressure in regions that contain gaps. In particular, it is clear that our method does not indiscriminately call gapped regions as type I sites. We cannot be sure that in this one case we did not get the right answer for the wrong reason and note that the use of PAML substitution counts and Gu's method for regions containing gaps merits further study.

### Seven LSCs were found in the Fpg/Nei protein family

Seven LSCs were identified in the Fpg/Nei family (Table 2). These include the well-known zinc finger, LSC6, which has four perfectly conserved cysteines in the BaFpg1, BaFpg2, actinomycetes (AcNei1 and AcNei2), PrFPG and MeNeil3 clades that coordinate the zinc (MeNeil2 possesses a CHCC zinc finger). However, the aligned positions are highly variable in the PFNei and MeNeil1 subfamilies and therefore easily identified as Type I sites. Since the changes in rates for these four sites have the same direction (the pair of clades ordered by substitution rate) it is not immediately clear whether the evolutionary transition between proteins with or without a zinc finger involves the loss of the structural role provided by the zinc finger or whether the role is filled by some alternative structure. In fact, the PFNei and MeNeil1 subfamilies, as well as the Mimivirus enzymes have a zincless finger that does not bind zinc but plays the same role [65], [66], [72].

**Table 2:** Seven LSCs from the Fpg/Nei protein family.

LSC	Suggested Role	Amino acids distinguishing the states
1	Stability of the interaction between N174 in the Helix-Two-Turns-Helix motif and the phosphate associated with the damaged base	K160,D178 (1R2Y)
		R171 (1K3W)
2	Stability of the catalytic helix and/or key DNA binding/catalytic residue Gly59/Lys60	L4,E8,R57 (1R2Y)
		Unknown
3	Stability of key catalytic residue Gly59/Lys60	E137,R58,G135, L134 (1R2Y)
		Unknown
4	Intercalation loop [48] inserts into spot left by “flipped-out” base and contacts opposite base	D110,F108,R113,R112,F114 (1R2Y)
		N76,M77,Y78 (1K3W)
5	DNA binding amino acid	Y242,G243,R244 (1R2Y)
		Unknown
6	Zinc finger which holds key damaged base phosphate binding residue Arg274 [48], [65], [66]	C249,C252,C269, C272 (1R2Y)
		Zincless finger amino acids
7	β F-α10 loop [67]	G218-G233 (1R2Y)
		Unknown

**Figure 2:**
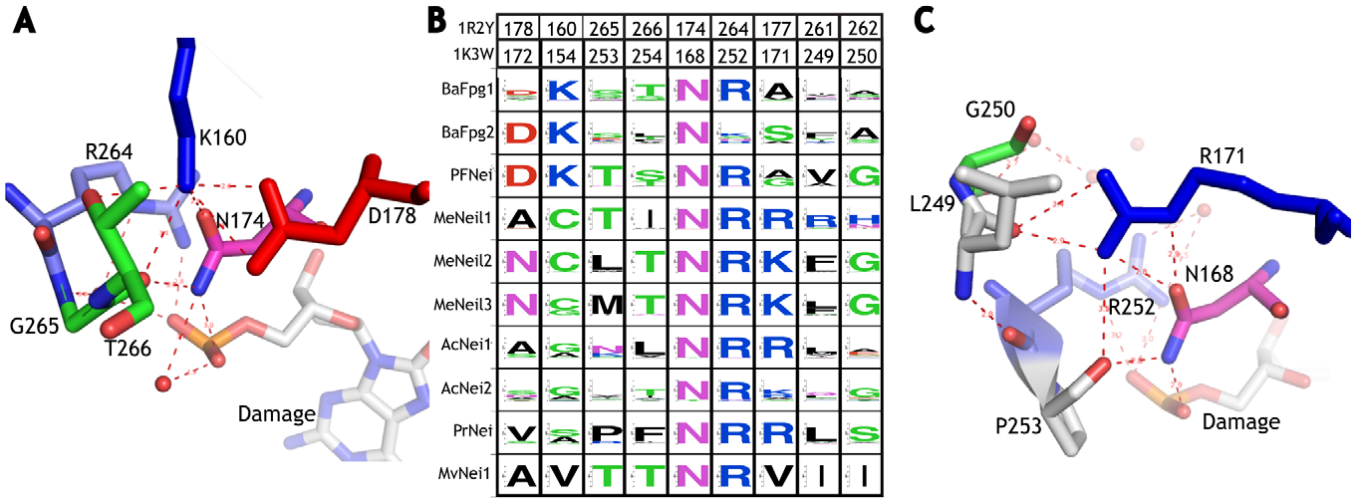
Multiple States of an LSC: Two solutions to the same problem. An LSC can have multiple states. A) State of LSC1 in the B. stearothermophilus MutM structure [67]. N174 (in pink), part of the helix-two-turn-helix (H2TH) motif along with two other amino acids (including the key amino acid R264, in blue) functions in the orientation and kinking of the DNA [70]. K160 (blue) helps keep the proper arrangement between the zinc finger and the H2TH [69]. B) Sequence logos for the each of the nine LSC1 amino acids in each of the three clades as well as MvNei1. Column headings indicate the aligned position in both the B. stearothermophilus MutM and E. coli Nei sequences. The sequence logos associated with 1R2Y K160 suggest that in three of the nine clades (BaFpg1, BaFpg2 and PFNei) the arrangement between the zinc finger and the H2TH is stabilized by a lysine in the same manner as in the B. stearothermophilus MutM protein. C) State of LSC1 in the E. coli Nei structure (62, PDB 1K3W). R171 hydrogen bonds to the other beta-sheet of the zinc-finger, presumably playing a role analogous to 1R2Y K160, which originates on a different helix. The sequence logos associated with R171 suggests that in six subfamilies (AcNei1 and AcNei2, PrNei and all vertebrate subfamilies), the arrangement between the zinc finger and the H2TH is maintained by an arginine or lysine in the same manner as in the E. coli Nei protein. For the subfamilies of BaFpg1 and PrNei, sites 160 and 266 are a type I, 174 and 264 are a type 0, and the rest are type II.

Other LSCs include Type I sites having different directions, which allows us, in addition, to identify alternative states of the character. LSC1 provides stability to N174 (Figure 2A) [67], part of the helix-two turns-helix motif. This residue orients and stabilizes the damaged base, along with R264 and K60, by hydrogen-bonding to P0, P-1 and P-2. BaFpg1, BaFpg2 and PFNei exhibit conservation of LSC1 amino acids D178 and K160 (Figure 2B), which form a salt bridge and stabilize N174 via a hydrogen bond (Figure 2A). However, in the other clades the corresponding amino acid substitution rates are significantly higher as a result of a change in the state of LSC1 (Figure 2C). Stabilization of other subfamilies is enhanced by a highly conserved arginine aligning with position 177 (Figure 2B,C; R171 in the E. coli Nei sequence), which is part of a network of hydrogen bonds that stabilize the critical asparagine (Figure 2C). The direction of the 171 Type I (Figure 2C) is opposite to that of D178 and K160 (Figure 2A), consistent with coupled compensatory changes. Both K160 and R171 have been mutated, resulting in significant loss of stability and activity [3], [12], [18], [19].

**Figure 3:**
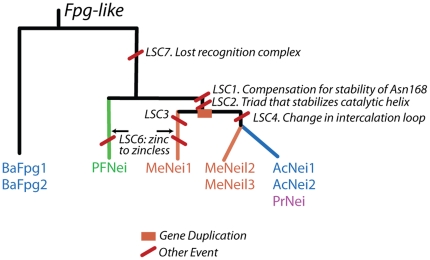
The states of LSCs can be used to infer the Fpg/Nei family phylogeny. The most parsimonious protein phylogeny consistent with the states of the six LSCs is shown with the changes in LSCs annotated as red bars. The choice of the root results in one of its children (BaFpg1, BaFpg2) represents well the diversity of bacteria while the other represents plants, fungi, and metazoans.

Since LSC admit some variation in amino acids their evolution is necessarily no faster than for single sites and they might be helpful as characters in a phylogeny of distant homologous subfamilies. We used maximum parsimony to construct a phylogeny of the nine clades using the LSCs as characters (Figure 3), reasoning that these higher-order structures would provide useful phylogenetic characters over long evolutionary time scales.

### Fpg/Nei Paralogous Clades Evolve at Different Rates

**Figure 4:**
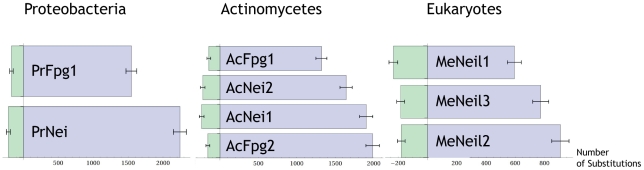
Rate variation does not differ dramatically between replicate Proteobacterium, Actinomycete, or Eukaryote organism tree topologies. Each column corresponds to one of the three organismal phylogenies. Each entry in a column (paired blue and green bars) represents an instance of the organismal phylogeny in the Fpg/Nei family protein phylogeny. The blue bars correspond to the number of substitutions from the last common ancestor (LCA) of each replicate tree to the present while the green bars correspond to the number of substitutions from the LCA of the phylogeny of replicate trees to the LCA of the each replicate tree.

Fpg/Nei has four actinomycetes subtrees, each composed of orthologous proteins from the same genomes [73], three subtrees from eukaryotes and two from proteobacteria. For each of these three sets of ortholog subtrees we examined variation in the protein-wide rate of evolution between member subtrees at two levels (Figure 4). First, we compared the number of substitutions in each subtree (since each subtree contains the same organisms the estimated number of substitutions represents the overall variation rate). Secondly, we estimated the branch length from the most last common ancestor of all subtrees to the root of each subtree.

**Figure 5:**
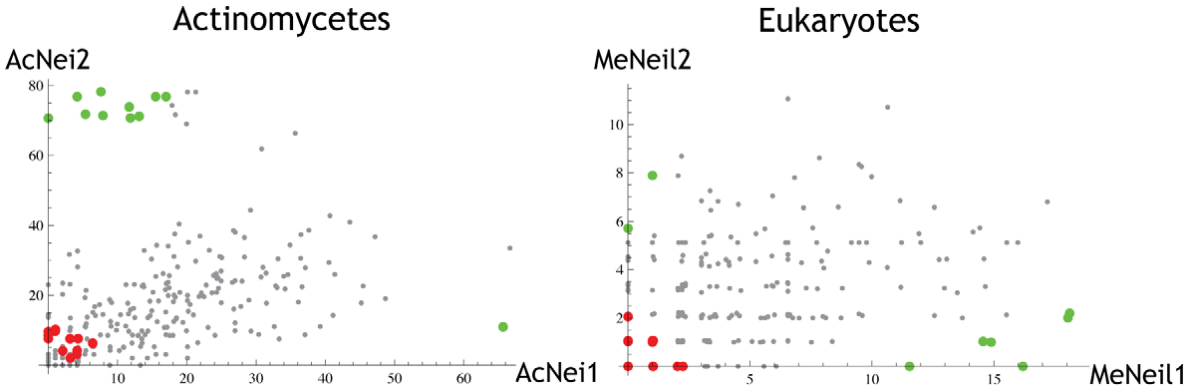
Substitution rates of individual aligned amino acid positions can differ between clades of orthologs. Substitution rates of individual aligned amino acid positions can differ between clades of orthologs from actinomycetes (left, Pearson correlation 0.47) or eukaryotes (right, 0.19). Each axis reflects amino acid variation rate in one of the replicate organism trees described in the legend to Figure 4. Each point is an aligned amino acid sequence position. Sites that have experienced a rate-shift (Type I) are green while those that exhibit an amino acid frequency-shift (Type II) are red.

For each of these six tests (one comparison for each of the 3 sets of subtrees, and one for the each of the three sets of ancestral edges leading to the subtrees, Figure 4) we were able to reject the null hypothesis that the summary substitution rate is uniform at p<0.03. BaFpg1 exhibits significantly fewer substitutions than the other paralogs in both actinomycetes (AcFpg1, p<10^−15^) and proteobacteria (PrFpg1, p<10^−15^). Likewise, MeNeil1 has significantly fewer substitutions than MeNeil2 and MeNeil3 (p<10^−15^). Interestingly, the edge leading to the MeNeil1 subtree is longer than for either MeNeil2 or MeNeil3 (p<0.003). While it is clear that the difference in substitution rates is statistically significant, the biological significance of differences of this magnitude is not clear. The rate variation shown in Figure 5 is nonetheless small compared with the substitution rates exhibited by other pairs of proteins and it seems unlikely that this variation is anomalous even among clades of paralogous proteins. It appears, therefore, that these proteins are all under significant selection pressure, even though some actinomycetes appear to have lost one member.

### Most amino acids exhibit changes in their structural or functional roles between paralogous clades

We can estimate the proportion of amino acids that have undergone functional divergence by calculating the type I and type II coefficients of functional divergence between the different subgroups [21], [25]. Type I coefficients refer to the proportion of sites that have undergone a change in rate between two clades (e.g. conserved in one clade, variable in another, a rate-shift [74]). Type II coefficients refer to the proportion of sites that have undergone a change in amino acid frequency (e.g. a conserved arginine in one clade vs. a conserved leucine in the other [25]). We found that all comparisons exhibit a coefficient of functional divergence between 0.4 and 0.8, implying that a large number of sites have changed in rate or constraint throughout evolution (Table 3).

**Table 3:** Coefficient of Type I (above diagonal) and Type II (below diagonal) functional divergence for Fpg/Nei clades.

	MeNeil1	MeNeil2	BaFpg1	BaFpg2	PFNei	MeNeil3	AcNei1	AcNei2	PrNei
MeNeil1		0.44	0.72	0.75	0.73	0.78	0.72	0.84	0.71
MeNeil2	0.7		0.51	0.63	0.5	0.57	0.68	0.54	0.44
BaFpg1	0.6	0.62		0.45	0.56	0.53	0.75	0.45	0.56
BaFpg2	0.74	0.67	0.28		0.62	0.67	0.7	0.58	0.56
PFNei	0.57	0.64	0.35	0.5		0.7	0.77	0.68	0.67
MeNeil3	0.74	0.54	0.62	0.7	0.77		0.71	0.53	0.46
AcNei1	0.71	0.66	0.39	0.56	0.65	0.69		0.49	0.6
AcNei2	0.72	0.66	0.45	0.53	0.67	0.62	0.36		
PrNei	0.71	0.7	0.56	0.58	0.73	0.71	0.56	0.49	

## Discussion

### Clustering of type I sites

We found with high statistical significance (p<0.002) that highly ranked type I sites in a quartet cluster in a loop that plays a role in function in one clade that it does not play in other clades. This suggests that the study of the function and evolution of the enzyme family might benefit from employing explicit random variables that represent concerted transitions because doing so should allow us to 1) identify groups of amino acids that cooperate in structure or function, and 2) derive phylogenetic characters that change in state more slowly than characters associated with individual sequence positions. Such phylogenetic characters might be suitable for resolving the deep branches of protein superfamilies. In this paper we address these two goals, opening the door for further development of methods for automatically finding LSCs. An important question is whether this clustering of rate-shift sites is a general aspect of protein families, a reasonable assumption that should be explored in future studies.

### LSCs Provide a Surprisingly Comprehensive Description of the Substrate Binding Site

**Figure 6:**
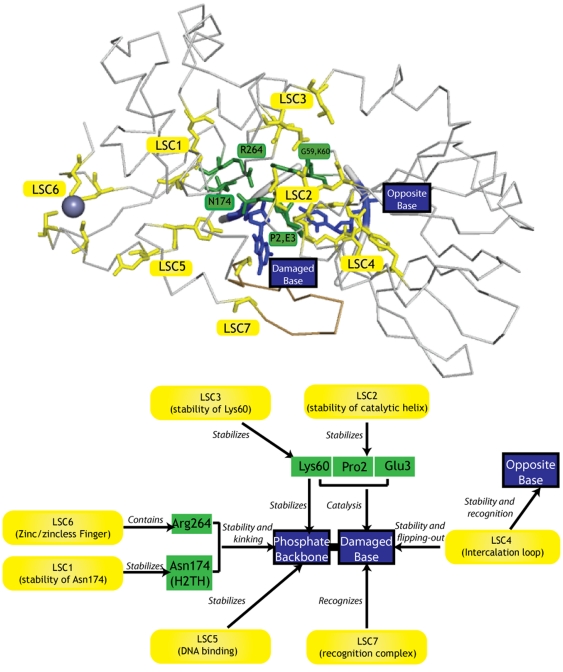
LSCs supply or stabilize residues that participate in enzyme-DNA interactions. Top) Amino acids side chains associated with LSC 1–6 are shown in the context of the protein backbone, DNA backbone, damaged nucleotide, opposite nucleotide, and Zn ion [67]. The green residues in both the structure (top) and the diagram (bottom) correspond to first-shell amino acids conserved in the entire family: R264 (contained in LSC6), N174 (stabilized by LSC1), and K60 (stabilized by LSC3/LSC2) stabilize the phosphate of the damaged base, and P2, E3 and are part of the catalytic residues [97]. The helix containing P2 and E3 may be stabilized by LSC2 as well. The enzyme everts the damage, and an intercalation loop (LSC4) fills the void and makes contact with the opposite base [68]. The damage itself in BaFpg1 is recognized by a recognition complex [67]. Other important residues not included here include H74 [97] and E6 [48]. A DNA binding residue not discussed in the literature corresponds to Tyr242 (part of LSC5).

Amino acids in Fpg/Nei family members directly contact the DNA substrate through interactions with the damaged base, opposite base, adjacent bases or phosphate backbone (Figure 6). Identification of highly conserved amino acids usually serves to find these first-shell amino acids [70], [75], [76], [77], [78], [79], [80]. Collections of second- and third-shell amino acids stabilize first-shell amino acids. We find that our methods for identifying LSC, which are based on differential conservation/variation rather than simply conservation, serve to find collections of second- and third-shell amino acids. In principle, these residues need not appear in LSC, that is, they might either be uniformly conserved or highly variable. However, seven LSC provide a surprisingly complete description of enzyme-substrate interactions.

More generally, we showed (Table 3) that a great percentage of amino acids change in rate and/or amino acid frequency at some point during evolution. Therefore amino acids not involved in substrate binding/catalysis are also found to exhibit changes in role and the possibility exists that LSC may provide a comprehensive description of enzyme structure and function more generally. These observations support a broadening recognition that quantitative studies of variation in selective constraints *within* aligned amino acid positions have the potential to add much to the picture provided by studying only structure or conservation.

### LSCs can serve as phylogenetic characters

For phylogenetic inference the time scale of character variation should match the evolutionary time scale in question. Nucleotide substitutions occur more rapidly than amino acid substitutions, which in turn occur more rapidly than changes in amino acid properties [81], [82], [83], [84]. It is therefore common to evaluate differences in amino acid properties, which may reflect changes in roles in structure and function that are latent in sequence alignments and which change relatively slowly. An obvious extension of this idea is that selection can act on the collective properties of a group of cooperating amino acids (example, a catalytic unit, [85]. Furthermore, changes in amino acid roles may be manifest as changes in substitution rates, not simply frequency. The rate of LSC evolution is necessarily similar to, or slower than, the variation rate of individual amino acids. The observation that LSCs are widespread suggests that our method for identifying LSCs provides useful characters for phylogenetic inference.

The deep branches in the phylogeny of the Fpg/Nei have been very difficult to resolve. Heterotachy, or within-site rate variation, is a known property of proteins [63] and makes conventional methods very difficult to apply [86], [87], [88]. Cheng and coworkers [89] suggested removing this difficulty by disregarding all heterogeneous rate amino acids. However, if we excluded these sites from our analysis we would be left with 10–20 amino acids which have not experienced a rate-shift, too few for a reliable phylogeny. Moreover, our approach suggests that those heterogeneous amino acids can provide valuable information regarding the deep branches of the phylogeny.

Our method produced a phylogeny consistent with reliable parts of existing phylogenetic trees, and resolved an existing branch (Figure 3). An important question pertains to the homology of the zincless fingers. Both the PFNei clade and the MeNeil1 clade have a zincless finger structure [49], [65] which are not similar in sequence. However, the resulting phylogeny, and in particular, the fact that the states of LSC1 and LSC2 are shared by BaFpg1, BaFpg2 and PFNei, whereas MeNeil1 shares a alternative state with the rest of the subfamilies supports separate origins of both zinc-less fingers.

We also used LSCs for gene classification [64]. Two viral proteins contain an Fpg-like gene [66]. We can use the presence/absence of the LSCs as a classification tool, determining that MvNei1 arose sometime after the divergence of MeNeil1 and PFNei, but not within MeNeil1. This we determine by noticing that MeNeil has a unique character state (LSC3) not shared by any other group of enzymes, whereas MvNei1 has the alternative character state that is shared with the rest of the subfamilies.

### Non-randomness of the Recognition Loop

The βF-∝10 loop plays a critical role in substrate recognition in the BaFpg1 subfamily [67], [68]. Even though the structure is known for some of the subfamilies: a helix in the human Neil1 structure [65], a longer loop in Mimivirus [72], but disordered in the E. coli Nei enzyme-substrate complex structure [70], its role is unknown in every clade except BaFpg1. Within BaFpg1, the conservation is not high, consistent with the observation of Fromme and Verdine [67] that damage recognition is provided by main chain amides. However, this loop is non-random in its distribution of amino acid rates.

### What is the basis for substrate specificity?

One of the most puzzling problems in base-excision repair enzymology is to understand how an individual member of the Nth or Fpg/Nei families excises structurally very different damages while failing to excise normal bases, which may appear relatively similar to some of the damages. Had we found precisely one LSC that 1) is comprised of amino acids near the damaged base and 2) has one state for each substrate specificity class then the interpretation would have been straightforward. In contrast, we found that LSCs essentially cover the protein-DNA interface. Furthermore we expect that a more comprehensive analysis of Type I and Type II sites remote from the protein-DNA interface will yield additional LSCs. LSCs appear to be more the rule than the exception.

Birth, death, and state transitions of LSC can be the result either of selection or drift. It may be that, like the majority of amino acid changes, LSC evolution is largely driven by drift. In this case LSC, like amino acid substitution, may ultimately prove more useful for understanding protein structure and evolution than in understanding specificity. On the other hand, the evolution of many of the LSC in the Fpg/Nei family may be the product of a highly complex selection for the ability to excise one diverse members of set of damaged while failing to remove the four normal bases. In contrast to cases in which a small number of key interactions between enzyme and substrate functional groups explains specificity, explaining specificity in the Fpg/Nei family may require the relative stabilities of damages in B-DNA as well as in metastable and transition states of the protein/DNA/solvent complex along multiple reaction paths that account for substrate diversity. For example, structural variation associated with rigid body movements of large portions of the protein suffices to give the state of an LSC an impact on specificity over long ranges. This is also consistent with the suggested plasticity of proteins that can be explained by population dynamics, and which results in different amino acid sequences giving rise to equivalent proteins in structure and function [33].

Nonetheless, some of our LSC are consistent with a link to specificity, for example, LSC5 in PFNei. LSC5 contains R/K244, GQR243 and Y242, which binds DNA. The function of Y242 is unknown but, while it is highly conserved at least in its aromatic character in most enzymes, it differs between plants and fungi. The Arabidopsis works best as an AP endonuclease and prefers oxidation products of 8-oxoguanine guanidinohydantoin (Gh), and spiroiminodihydantoin [49]. The absence of this LSC, very well conserved in the rest of the family, might give insight into its workings.

In summary, analysis of LSC provide a novel and powerful way to describe protein evolution over time scales for which amino acid substitution models weaken. We propose that improved methods for automated identification of LSCs in combination with quantitative models of the birth, death, and state transitions of LSC will improve our understanding of protein structure, function, and evolution.

## Materials and Methods

### Estimation of Fpg/Nei subfamily phylogenies

Fpg/Nei homologs were identified in the NCBI RefSeq database [90] using the PFAM [91], [92] domain profile pfam06831 and the CDD database [93] search software. T Coffee [94], [95] was used to identify a smaller set (415) of sequences that represent the diversity of the tree. MAFFT [96] and ASH [92] were used, with high accuracy parameters (iterative refinement incorporating local alignment information for the alignment), to align the sequences based on crystal structures [65], [67], [69], [97], [98]. PFAAT [99] was used for visualization of the sequence alignment and Seaview [100] was used to remove phylogenetically uninformative sites. Neighbor-joining phylogenetic trees were constructed using PHYLIP [57] PROTDIST, NEIGHBOR (standard parameters) and SEQBOOT (100 bootstrap replicates). Sequence alignment, genbank ids and trees are available upon request.

Based on the resulting sequence phylogeny, as well as on taxonomy and biochemical considerations, nine subfamilies were identified (Table 4). CDTree [93] was used to remove false positives and to build a position-specific scoring matrix for each subfamily. Subsequently, these models were used to subclassify RefSeq Fpg/Nei hits using RPS-BLAST [54] (see proteopedia page for the resulting table). Sequences were removed if they attached to a tree with an edge representing more than one substitution per site on average. Maximum likelihood trees, used exclusively in what follows, were constructed using RAXML [53].

**Table 4:** Features of the subfamily alignments and trees.

Subfamily	Membership	Tree Length (mean substitutions per site)	Specificity Loop Length (amino acids)	Total Length1 (amino acids)	Number of sequences
MeNeil1	Metazoans, e.g. hNEIL1	13.6	18	390	34
MeNeil2	Metazoans, e.g. hNEIL2	7.4	10	332	21
MeNeil3	Metazoans, hNEIL3	6.1	12	605	22
BaFpg1	Most bacterial Fpg	41.9	23	269	62
BaFpg2	Narrow bacterial distribution of Fpg that includes Actinomycetes	18.1	18	288	52
PFNei	Plants/Fungi	16.8	12	390	45
AcNei1	Actinomycetes, e.g. MtNei1	19.2	25	268	53
AcNei2	Actinomycetes, MtNei2	18.2	16	265	55
PrNei	Proteobacterial Nei	7.6	17	263	71

1 Length of the reference sequence used to calculate rates for each subfamily.

### Estimation of numbers of substitutions

PAML [55] was used to estimate the number of substitutions at each site in each subfamily. Briefly, PAML infers ancestral states and, for each site, counts edges associated with different end states. The advantage of this method is that it uses branch length information as well as the substitution rates between the amino acids to calculate the ancestral states [55]. The correction of Gu [101] was then applied to these counts to obtain the estimated number of substitutions. PAML assigns amino acids, not gaps, to all ancestral sites. As a result each gap in an extant sequence is associated with one inferred substitution so, for example, an aligned position having N gaps is assigned at least N substitutions.

### Identification of Type 0, I and II sites associated with each pair of subfamilies

Classification of sites into Type 0 (highly conserved), Type I (undergo a change in substitution rate along an edge) and Type II (undergo a change in preference for amino acid properties) have been described by Gu [21]. The maximum likelihood methods of Gu et al. [21], [22], [23], [24], [25] were used to identify type I and type II sites based on a posterior probability greater than 90%. Mathematica [102] was used for the implementation of those methods and for all calculations and graphs (software available upon request). Briefly, identification of type I sites is based on application of a likelihood model to two subfamilies; each site is either related to functional divergence or not [3]. In the former case substitution rates are unequal while in the latter case substitutions rates are equal.

Based on the way PAML counts substitutions for positions containing gaps, positions having a high proportion of gaps are assigned high substitution rates. Thus, although we did not implement a formal probability model for gaps, the resulting substitution rate assignments nonetheless reflect selection pressure that is less than for ungapped positions. This gives the desirable feature that segments that are highly conserved in one subfamily but gapped in another subfamily are easily identified as segments of type I sites. In principle the possibility exists that this method assigns type I calls when part of the protein is weakly conserved in one subfamily and gapped in another. We inspected our type I calls and found that in nearly all cases the slower rate in the rate pair is below the median substitution rate.

Type II sites were identified using 1) substitution counts for each subtree, 2) PAML's ancestral reconstruction at the subtree roots, and 3) a definition of radical changes between clades. We considered a change radical if it altered membership with respect to four groups: charge positive (K, R, and H), charge negative (D and E), hydrophilic (S, T, N, Q, C, G, and P), and hydrophobic (A, I, L, M, F, W, V, and Y) [25]. We implemented both methods of Gu et al. and applied it to multiple clades. Our results were checked by comparison with Gu's program Diverge [103].

### Statistical test for the structural clustering of type I sites

We designed a test to determine if the sites that change in rate (type I sites) cluster together in space. Consider the quartet ((A,B),(C,D)). Our test statistic, the number of pairs of type I transitions that occur on the same edge and within 4 angstroms of each other, is large when changes in selection pressure involving neighboring amino acids tend to be concerted. We ranked all sites based on the posterior probability that they exhibit Type I transitions along (A,B) and, separately, along (C,D). The value of the test statistic was calculated using the top N such sites for each edge. The distribution of the test statistic under the null hypothesis was determined by permuting the edge assignments of the 2N Type I transitions.

One consideration is that, even under the null hypothesis, type I sites are not expected to be distributed uniformly on the protein. Type I sites may be spatially correlated because changes in rate tend to happen on specific regions of the enzyme. We therefore condition on observed type I sites, permuting type I events among edges in the phylogeny.

A second consideration pertains to the thresholding procedure used to identify type I sites associated with different pairs of clades. The permutation test requires control over both false positive and false negative rates for both edges. We find that, with one poserterior probability threshold, it is not possible to achieve acceptable false positive and false negative rates for both edges. We handle this problem by choosing the same number, N, of type I sites for each clade (see below). We do not consider clades having a tree length so small that they are unlikely to exhibit N type I sites.

Based on the considerations above, we consider quartets such as ((BaFpg1,PFNei), (AcNei1,AcNei2)) that contain large numbers of substitions, both within and between clades. We chose the 25 type I sites for each of the two edges having the lowest posterior probability (common type I sites are discarded). The (BaFpg1, PFNei) and the (AcNei1, AcNei2) edges having 13 and 18 neighbor type I pairs, respectively, for a total of 31 for our statistic. They have 3 type I sites in common, thus we will then do a statistical permutation test in which we resample without replacement pairs of 22 sites from our pool of 44, and calculate our statistic. Other quartets exhibited a similar pattern (results not shown). A second statistic was used to determine the importance of the structure on the clustering, and consisted of the number of pairs of amino acids that are next to each other. There were 12 consecutive pairs in total for the statistic.

### Identification of LSCs based on Type class assignment and structural information

Consider the set, V(A,B), of amino acid positions assigned to class Type 0, I or II with respect to a pair of subfamilies (A,B). Consider also the set E of pairs of elements of V having α-carbons within 4 angstroms. An LSC is a connected sub graph of the graph (V,E) containing at least two amino acids, at least one of which is either Type I or II. We found V associated with every pair of the 9 subfamilies. All programming was done in Mathematica 6.0 and 7.0, and the reference structure was the Bacillus stearothermophilus MutM (PDBid 1R2Y) [67]. All structure visualization was done in PyMOL [56]. In principle different structures would give different results, but for the family we are interested in the structures are so conserved that we expect the results to be highly similar.

### Evaluation of the uniformity of selection pressure on sets of amino acid residues

Given a set of aligned positions, α, and a set of trees, τ, substitution counts, 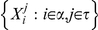 and tree lengths, 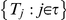, can be used to test the null hypothesis that selection pressure is uniform among trees or among sets of aligned positions.

Consider the BaFpg1 recognition loop, the sequence between G218 and L239 in the Geobacillus stearothermophilus Fpg sequence gi38492995. We tested the null hypothesis that amino acids in the loop represent the selection pressure in the remainder of the protein. In this case there is only one tree length, so we are comparing two sets of substitution counts 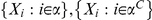. More precisely, we tested the null hypothesis that the two distributions have the same location, using the mean as a test statistic, as well as the null hypothesis that the two distributions are equal, using the sum of ranks. In both cases the null distribution was obtained by resampling (100,000 samples).

Sums of Poisson processes follow a Poisson distribution, so we test the hypothesis that the summary Poisson rate parameter, λ, is uniform across a sets of n trees (H_0_),
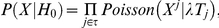
where 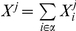, versus the alternative hypothesis that there are different rates (H1),
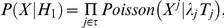
Under the null hypothesis the likelihood ratio test statistic, −2 Log Λ, where
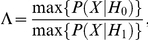
is distributed as chi-square with n−1 degrees of freedom. We used this likelihood ratio to test the null hypothesis that the recognition loop is under uniform selection pressure among the nine subfamilies.

Consider 1) a sequence tree consisting of an n-ary root node, each child of which is a tree having the same topology and m leaf nodes associated with the same set of genomes, and 2) an alignment of the associated n×m sequences represented in the tree. We used the likelihood ratio statistic to test the null hypothesis the Poisson rate parameters associated with 1) the subtrees or 2) the edges connecting subtrees to their common ancestor, are equivalent.

### Comparison of the summary evolutionary variation rate between subfamilies

#### Proteopedia page

The Proteopedia website software [51] was used to provide interactive three-dimensional representations of the LSCs as well as additional information on the Fpg/Nei protein family, including site-directed mutagenesis experiments and distribution of homologs among taxa.
